# Current paediatric orthopaedic practice in hereditary multiple osteochondromas of the forearm: a systematic review

**DOI:** 10.1051/sicotj/2018002

**Published:** 2018-03-21

**Authors:** Tamer A. EL-Sobky, Shady Samir, Ahmed Naeem Atiyya, Shady Mahmoud, Ahmad S. Aly, Ramy Soliman

**Affiliations:** 1 Division of Paediatric Orthopaedics, Department of Orthopaedic Surgery, Faculty of Medicine, Ain-Shams University, Abbasia, Cairo Egypt; 2 Division of Hand Surgery, Department of Orthopaedic Surgery, Faculty of Medicine, Ain-Shams University, Abbasia, Cairo Egypt

**Keywords:** Children, Hereditary multiple exostoses, Multiple cartilaginous exostoses, Diaphyseal aclasis, Benign forearm tumours, Skeletal dysplasia, Ulna lengthening

## Abstract

*Introduction*: This systematic review aims to answer three research questions concerning the management of hereditary multiple osteochondromas of forearm in children: What is the best available evidence for the currently employed surgical procedures? What patient characteristics are associated with better prognosis? What disease characteristics are associated with better prognosis?

*Methods*: We searched the literature using three major databases with no publication date restrictions. To enhance search sensitivity and maintain precision we used keywords/subject terms correlating with patient population, problem and interventions. We used strict inclusion/exclusion criteria to improve validity evidence.

*Results*: The search process yielded 34 eligible studies with a total of 282 patients (315 forearms). We comprehensively analysed study and patient demographics and interventions and outcomes. Eleven studies (32%) had a long-term follow-up and 31 studies (91%) were retrospective. Of the total number of forearms, ulnar lengthening +/− associated procedures was used in 210 forearms (66.7%), isolated osteochondroma excision in 65 forearms (20.6%) and isolated distal radius hemiepiphysiodesis in 15 forearms (4.7%) among others.

*Discussion*: Ulnar lengthening can restore radiologic anatomy, improve appearance and to a lesser extent objective clinical parameters like joint range of motion on the short/intermediate term. Isolated osteochondroma excision can relief pain and satisfy cosmetic concerns occasionally. There is poor evidence to suggest that surgery improves quality of life or function. Predictors of surgical success in regard to patient and disease characteristics remain elusive. Natural history and prospective randomized control studies where the control group receives no treatment should be rethought. They have the potential for bias control and identification of the ideal surgical candidate. The complex interplay between the confounding variables has undermined the capability of most studies to provide well-grounded evidence to support and generalize their conclusions. Valid quality of life scales should supplement objective outcome measures.

## Introduction

Hereditary multiple osteochondromas (HMO) are uncommon benign bone tumors. They are usually diagnosed in early childhood. HMO are inherited in an autosomal dominant manner. The two genes in which pathogenic variants are known to cause HMO are *EXT1* and *EXT2* [1,2]. The pathogenesis is linked a critical reduction in heparan sulfate chain elongation [2]. Contrastingly, solitary osteochondromas are common benign bone tumours. The growth pattern of a solitary osteochondromas is comparable to that of HMO. The diagnosis of HMO is based upon a distinct clinical and radiographic appearance. A fundamental clinical feature is multiple firm swellings erupting from the ends of long bones or from the surface of flat bones usually symmetrical [1]. Radiologically they present as multiple juxtaphyseal cartilage capped bony growths with undisturbed course of cortex and medullary bone from the normal bone into the osteochondroma [1,2]. Masada and colleagues [3] classified HMO into three main groups based upon the pathologic anatomy. Patients with HMO can exhibit limb length discrepancy, angular deformities around the knee, ankle and forearm, short stature, painful joint range of motion, joint subluxation and neurovascular compression [1,2]. Up to 70% of patients with HMO manifest in forearm deformities [4]. Most forearm and hand deformities are clinically pronounced [4,5]. Unbalanced physeal growth between the radius and ulna can result in forearm bowing, relative shortening of radius or ulna, carpal instability, and radial head dislocation with subsequent limitation of forearm rotation [1,4]. Forearm HMO has been managed by one or more of the following procedures isolated excision of osteochondroma [6–12], acute [7,12,13], and/or gradual [6,14–26] ulnar lengthening, combined ulnar and radial lengthening [27], distal radial hemiepiphysiodesis [3,12,28,29], corrective radial osteotomy [7,13,30], creation of one-bone forearm [31–33], radial head relocation [8,10,22], reconstruction of the distal ulnar epiphysis by vascularized proximal fibula epiphysis [34] and Sauve-Kapandji procedure [10]. Nevertheless, the optimal management of HMO of the forearm is greatly disputable. The best evidence for each of the practiced surgical procedures, the optimal timing for intervention and predictors for surgical success are alike unsettled disputes. Some authors cast fresh doubts about the value of surgery in regard to improving function [7,35]. This topic has not been critically appraised before in the literature. This systematic review aims to resolve the above-mentioned disputes. In consequence we formulated the following research questions relating to the management of HMO of forearm in children: 1) What is the best available evidence for surgical procedures used to manage HMO? 2) What patient characteristics are associated with better prognosis? 3) What disease characteristics are associated with better prognosis?

## Methods

### Search approach

**Figure 1:**
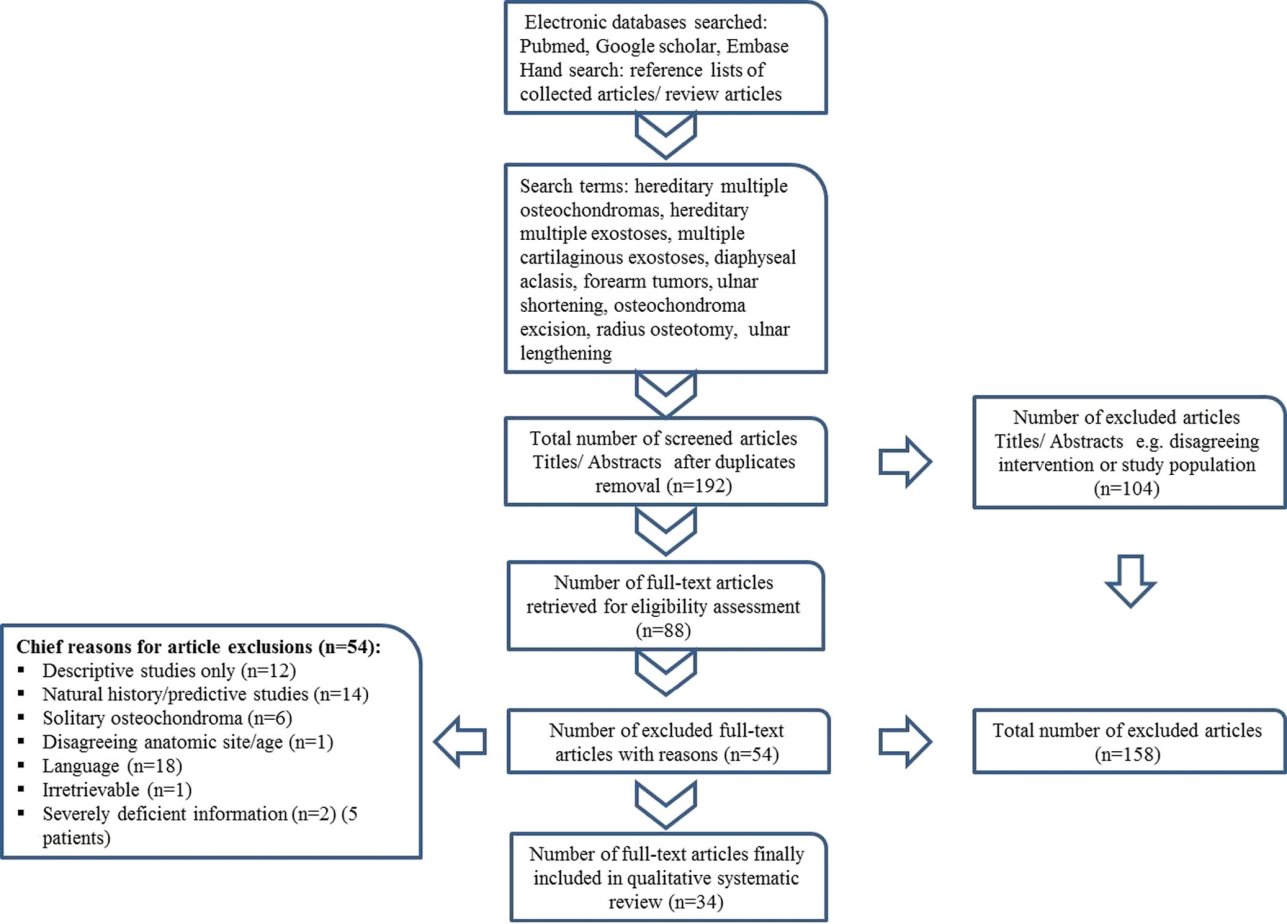
A schematic representation of literature extraction process.

This article does not contain any studies with human participants or animals performed by any of the authors. All authors shared in the study selection and data extraction process relating to the surgical management of HMO of the forearm in children. We conducted a search for English language publications before July 2017 employing the following electronic databases PubMed, Google scholar and Embase. We checked the reference lists of the captured articles and review articles for additional eligible publications. We also screened articles that cited the captured articles. We discarded non-peer reviewed literature that was not published in scientific journals and secondary research such as review articles, letter to the editor and commentaries. We conducted the initial search on May 2017. We performed an additional search prior to manuscript submission to make certain the extracted literature is updated. To expand the recapture of relevant studies our search strategy comprised both keywords and index words in accordance with Medical Subject Headings. We used Boolean operators properly to optimize search results quantitatively and qualitatively. The three main Boolean operators are AND, OR, and NOT. Boolean operators are used to narrow, broaden or restrict the search results. We aimed at avoiding biased inclusion terms. Hence, the selection of search terminology was subdivided according to (a) patient population, (b) problem and (c) intervention terms. We retrieved relevant studies using the following patient population and problem terms: children, paediatric, hereditary multiple osteochondromas, hereditary multiple exostoses, multiple cartilaginous exostoses, diaphyseal aclasis, forearm tumors, ulnar shortening. Additionally, we used the following intervention terms: osteochondroma excision, radius osteotomy, ulnar lengthening, distraction osteogenesis. We did not impose limiting terms with regard to study design types. The collected studies were excluded as follows; (a) descriptive studies reporting the clinical and/or radiologic features, (b) studies reporting the natural history, (c) studies reporting on adults, (d) studies reporting exclusively on solitary osteochondromas, (e) studies reporting solely on pathologies other than HMO, (f) studies with follow-up < one year, (g) HMO managed in the context of malignant transformation and (f) studies reporting solely on HMO of the hand. We included prospective and retrospective studies. We also included case series and case reports. If studies were heterogenous for age population and pathologic disorder, only skeletally immature patients with HMO were selected. Disputes in regard to study selection were settled with face-to-face meetings. A schematic representation of the literature extraction process together with exclusions is provided (Figure 1).

### Quality appraisal instruments

The reported items of this review were in concordance with the Preferred Reporting Items for Systematic Reviews and Meta-Analyses statement [36]. We used the systematic review critical appraisal worksheet from the University of Oxford Centre for Evidence-based Medicine www.cebm.net/critical-appraisal to check quality through all phases of this systematic review [37]. We identified factors that may reflect a significant research bias before, during and after the conduction of included studies with respect to patient selection, outcome measure assessment, statistics and confounding variables. We employed a valid instrument designed to evaluate the methodological quality of non-randomized surgical studies, whether comparative or non-comparative (MINORS) [38]. We selected MINORS [38] evaluation instrument as case series observational studies were the key of the primary studies. We conducted a comprehensive comparison between the aggregated treatment outcomes and tried to identify patient and disease features linked to better prognosis. To avoid bias in favour of reporting positive findings only we decided to finalize our systematic review protocol in advance of any data extraction.

## Results

### Study demographics

The literature extraction process involved 4 phases: (a) identification, (b) screening, (c) eligibility, and (d) inclusion. There were only three prospective case series studies [20,27,39] (OCEBM type II) versus 31 retrospective studies (OCEBM type III). These prospective studies had small sample sizes [39] or were simply case reports [20,27]. The characteristics of the 34 final studies included in this systematic review are presented in (Table 1). Eleven studies (32%) had a follow-up > 5 years, five of which were relatively sizable with study participants ≥ 10. Of the 34 included studies, 32 were published in recognized orthopaedic society journals. The publication years ranged from 2016 to 1984. Eight were multicentre studies [8,11,13,15–17,19,23]. According to MINORS the methodologic quality all but three prospective studies scored 5 out of 8.

**Table 1:** Study demographics.

Author:Year [Reference]	Patients (N)	Forearms (N)	Male:Female (N)	Mean age (Y)	Mean follow-up (Y.M)	Family history (N)	Surgical indications	Masada subtype
Cho:2014 [19]	3	3	0:3	7.2	2.1	NR	Restricted rotation & cosmetic	IIB (3)
D'Ambrosi:2016 [15]	15	15	8:7	10.1	6.4	NR	Restricted rotation or daily activities, ulnar shortening ≥ 1.5 cm	IIA (6), IIB (8), III (1)
Demir:2011 [39]	6	6	2:4	12	4.2	NR	Restricted rotation, pain, neurovascular compression & cosmetic	IIA (3), IIB (3)
Vogt:2011 [21]	12	12	3:9	9.8	2	NR	Ulnar shortening ≥ 1 cm, progressive deformity, functional impairment	I (7), IIA (1), IIB (4)
Tang:2013 [41]	14	14	8:6	9.2	3.6	NR	Restricted daily activities, Ulnar shortening ≥ 1.5 cm, & cosmetic	I (14)
Masada:1989 [3]	11	13	5:6	10.3	2.6	7	Restricted daily activities & ROM	I (9), IIA (1), IIB (2), III (1)
Beutel:2014 [20]	1	1	1:0	11	2	None	Pain & restricted ROM in elbow	I (1)
Hill:2011 [22] #	4	5	2:2	8.8	2.2	NR	Wrist, forearm or elbow deformity with ulnar or radial shortening > 2 cm, particularly young children, restricted daily activities	NR
Litzelmann:2012 [7]	14	15	NR*	11.1	9.8	NR	Cosmetic or functional (pain or limited mobility) based upon surgeon preference	I (7), IIA (1), IIB (4), III (3)
Jiya:1997 [11]	10	12	NR	13.3	6.3	NR	Restricted daily activities, grip strength	I (9), III (3)
Shin:2006 [10]	22	22	NR	9.2, 8.8, 11.1 †	3.6	NR	Pain, functional loss of movement of the forearm & cosmesis.	I (11), I and/or IIB (11)
Rasool:2008 [31]	2	2	0:2	5	1.3	NR	Elbow pain & deformity	IIA (1),IIB (1)
Pritchett:1986 [26]	8	10	3:5	11.4	3.1	NR	Ulnar shortening ≥ 1.5, carpal slip ≥50%, symptomatic radial head instability, restricted daily activities & cosmetic.	NR
Massobrio:2015 [27]	1	1	0:1	9	20	NR	Functional restriction of ROM in elbow & wrist	I (1)
Kelly:2016 [28]	13	15	NR	Boys:10.3 & Girls: 11.5	5	NR	NR	NR
Tonogai:2015 [18]	2	3	1:1	9.5	3.7	NR	Restricted ROM	I (2), III (1)
Refsland:2016 [14]	17	17	11:6	7	4.6	NR	Restricted ROM	I (10), IIB (7)
Bauer:2017 [17]	3	3	NR*	15	NR	NR	Restricted ROM	NR
Yang:2013 [34]	2	2	1:1	6.5	Boy:1 & Girl:8	1	Deformed wrist	I (1), IIB (1)
Bilen:2009 [23]	7	8	3:4	10	3.3	NR	NR	I (5), IIB (3)
Mader:2003 [40]	2	4	1:1	8.8	2	NR	Functional restricted of ROM, ulnar/radial shortening ≥ 2 cm, carpal slip ≥ 50% & RAA ≥ 40°	I (4)
Peterson:2008 [32]	1	1	1:0	9.2	15	NR	Elbow deformity & unstable, forearm length discrepancy, restricted motion	IIB (1)
Eralp:2016 [16]	3	4	NR	10	5.8	NR	Deformity & restricted daily life activities	I (4)
Waters:1997 [13]	17	NR	11:11*	10.7*	3	NR	Progressive forearm/wrist deformity, limited/painful ROM, radial head subluxation & based on definite radiographic criteria	I (12), IIA (4), IIB (1)
Ip:2003 [30]	6	6	5:1	7.6	2.5	5	Daily life activities & cosmetic	I (6)
Song:2013 [6]	10	13	6:4	9.6	4.8	NR	Functional limitation of motion, pain & definite radiographic criteria	I (12), IIB (1)
Akita:2007 [8]	23¥	31	17:6*	11	12.8	18	Osteochondroma excision: painful ROM or cosmetic. Lengthening/osteotomy: based on definite radiographic criteria	I (21), IIA (2), IIB (3), III (5)
Matsubara:2006 [24]	7	7	3:4	10.8	7.1	NR	Radial head dislocation, daily life activities, painful forearm rotation, deformity & cosmetic.	I (6), IIA (1)
Ishikawa:2007 [9]	13	14	6:7	7.9	4.5	NR	NR	I (14)
Cheng:1991 [25]	4	4	2:2	12	1.5	NR	Ulnar shortening ≥ 2 cm, radial head instability & functional limitation of ROM	NR
Rodgers:1993 [33]	2	2	2:0	13.5	8.5	1	Painful radial head dislocation +/− severe elbow & forearm deformity	IIA (1), IIB (1)
Irani:1993 [43]	10	12	4:6	10.8	6.5	NR	Pain & cosmetic, progressive deformity (≥ 1 cm ulnar shortening).	I (4), IIA (4), III (4)
Fogel:1984 [12]	17	21	12:5	9	4.5	NR	Pain & cosmetic, progressive radiologic deformity (≥ 1.5 cm ulnar shortening, radial articular angle ≥ 30° & carpal slip ≥ 30°), rotation restriction & symptomatic radial head subluxation.	NR
Arms:1997 [29] ∞	22	NR	NR	Range 7–77 (mean NR)	> 2	NR	Symptomatic osteochondroma prominent, painful, aesthetically unacceptable.	NR

*N* number, *NR* not reported.

* gender distribution & other demographics were provided either for the overall number of children diagnosed with HMO and not for those operated, or for overall number of different diagnostic groups and not for HMO, or for both skeletally mature & immature;

# patient characteristics, follow-up, methodology, outcome and complications (except radial head status) were provided for 10 patients with various diagnostic groups and the details of HMO patients were not provided separately;

† a separate mean was given for each of he 3 surgical procedures conducted;

¥ 4/23 patients were skeletally mature;

∞ the study comprised a cohort of patients treated conservatively and surgically 37 of which were subjected to a telephone questionnaire but patient demographics, disease characteristics and interventions were not provided separately for operated group.

### Patient characteristics

The summed number of patients enrolled in the included studies was 304. One study with 22 patients was excluded from analysis due to poor demographic reporting but its implications were discussed [29]. Therefore, the final number of patients enrolled was 282 patients with 315 forearms. The mean age of patients for individual studies ranged from (5–13.5) years. One study provided a separate mean age for each of the three surgical procedures conducted [10] and another provided a separate mean age for boys and girls [28]. Gender distribution was provided for 222 patients (79%) of the 282 patients finally enrolled in the review. There were 118 (53%) males and 104 (47%) females. Six studies did not provide the gender distribution [7,10,11,16,28,29]. One study did not provide a mean age or follow-up and provided a range instead [29]. For publications that did not directly report the Masada types the information was extrapolated from descriptive clinical picture and/or radiographs. Interestingly, in one study [26] neither Masada types nor osteochondroma excision were reported. Of the 257 forearms that “reported” Masada types, 166 (64.6%) were type I, 25 (9.7%) were type IIA, 48 (18.7%) were type IIB and 18 (7%) were type III. Two studies [22,25] referenced Masada but did not provide details of patients' radiographic grading.

### Interventions and outcomes

A detailed reporting of the interventions, outcomes, and complications at final follow-up of the included studies in this review is presented (Table 2). The indications of surgical intervention expressed variability among studies. Restriction of daily activities and/or range of motion in forearm/elbow were the most common indications of intervention. Most studies tended to favour objective physician-reported clinicoradiologic data as an outcome measure. Contrastingly, the majority of subjective patient-reported rating scales/questionnaires were non-validated and statistical significance was inadequately implemented. One study [7] restricted the implementation of the patient-reported rating scale on the sub-category of patients subjected to gradual ulnar lengthening by an external fixator. This is seen as an effort to reduce the impact of confounding variables. All studies employed plain radiography as a principal diagnostic tool. Two studies [20,39] employed MRI as an adjuvant imaging modality one of which [39] used CT scan and bone scintigraphy in addition. One study [27] employed ultrasound to monitor callus progression. The vast majority of studies assessed the radiographic outcome in accordance with proposed measurements by Fogel and colleagues which are a widely reported [12]. Five studies [6,9–11,41] used another referenced radiographic measurement by Burgess and Cates [42]. Among the various radiographic measurements, radial articular angle, carpal slip, ulnar shortening, and radial head stability were the most commonly shared by included studies. Contrastingly, radial length was the least used measurement. Ulnar lengthening +/− an associated on demand procedure was conducted on 210 forearms (66.7%) of the summed number of forearms. Associated procedures included a combination of one or more of the following: angular correction, osteochondroma excision, corrective radial osteotomy distal radial hemiepiphysiodesis and open reduction of radial head. Of the 210 forearms (66.7%) subjected to ulnar lengthening, 145 forearms (69%) were performed gradually and 65 forearms (31%) were performed acutely mostly with bone grafting [3,7,8,10–13,26,30]. Of these later studies two used acute ulnar lengthening exclusively [12,13]. All “gradual” ulna lengthenings were performed by a uniplanar fixator except in 17 forearms (11.7%) a multiplanar fixator was used [6,19,20] and five studies with 22 forearms (15.2%) used both uniplanar and multiplanar [18,22–24,30]. All ulnar lengthening osteotomies/cortectomies were performed at a mid or proximal diaphyseal level. One study employed a simultaneous radial and ulnar lengthening in one forearm [27] and three others (seven forearms) conducted isolated radial lengthening as part of a series including ulnar lengthenings [8,18,24]. The vast majority of radial osteotomies were performed in association with ulnar lengthening procedures. Isolated corrective radial osteotomies were done in two forearms (0.5%) only [7].

**Table 2:** Interventions and outcomes.

Author:Year [Reference]	Operative procedure (forearms)	Preoperative radiography	Postoperative radiographyμ	Preoperative clinical tools	Postoperative clinical outcomeμ	Complications*
Cho:2014 [19]	Osteochondroma excision (ulna), gradual mid-diaphyseal ulnar lengthening + 5 mm overlengthening, multiplanar Ilizarov external fixator (3).	Radial articular angle, radial length, radial bowing, percentage of radial bowing, carpal slip, ulnar length, ulnar shortening, percentage of ulnar shortening & radial head stability	Statistically *non*-significant improvement of all indices	Patient-reported functional assessment scale, & ROM	All patients were satisfied, statistically non-significant improvement of ROM	Callus fracture, & asymptomatic resubluxation of radial head (1 each).
D'Ambrosi:2016 [15]	Osteochondroma excision (ulnas), gradual middiaphyseal ulnar lengthening, uniplanar external fixator (15), +/− distal radioulnar synostosis separation.	Radial articular angle, carpal slip, ulnar shortening, radial head dislocation, & relative ulnar shortening	Statistically significant improvement of all indices. Carpal slip remained in 3/7 cases.	Patient-reported functional assessment scale, ROM, MAYO Elbow Score, VAS, SF-12	All patient-reported and physician-reported parameters showed a statistically significant improvement.	Non-union successfully treated by bone grafting & compression plate fixation (1).
Demir:2011 [39]	Osteochondroma excision (2 ulnas, 1 radius), gradual mid-diaphyseal/proximal ulnar lengthening, uniplanar external fixator, +/− radial osteotomy (2).	Radial articular angle, carpal slip, ulnar variance, radial bowing. MRI, CT scanning and bone scintigraphy were also used	Significant improvement of all indices but statistical significance was not used.	Patient-reported assessment scales for daily life activities, pain, cosmetic, DASH & ROM	All patient-reported parameters & ROM improved and all patients were satisfied with their cosmetic outlook. Only one patient reported mild pain.	Callus fractures successfully treated by plating & intramedullary fixation (2).
Vogt:2011 [21]	Gradual diaphyseal ulnar lengthening, uniplanar orthofix external fixator (12), +/− osteochondroma excision (5 ulnas), +/− proximal radioulnar synostosis resection (1) +/− radial osteotomy (4).	Radial articular angle, ulnar shortening, carpal slip, radial head dislocation	Radial articular angle & ulnar shortening showed a statistically significant improvement. Carpal slip disappeared in 3/5 cases and asymptomatic radial head dislocation persisted in all 5 cases.	ROM only	Forearm: 41% improved, 25% deteriorated, 4% unchanged. Elbow & wrist flexion/extension: 84% unchanged, 1 case improved and another deteriorated. Wrist adduction/abduction: 59% improved & 41% unchanged.	Premature callus Consolidation successfully treated with repeat corticotomy (1).
Tang:2013 [41]	Osteochondroma excision (ulna), gradual distal ulnar osteotomy & lengthening +/− 5 mm overlengthening, uniplanar external fixation (14).	Radial articular angle, radial bowing, percentage of ulnar shortening, & carpal slip†.	Radial articular angle & carpal slip improved in all but 2 cases. Ulnar shortening was corrected in all cases.	Objective & subjective assessment of wrist function for: percentage of grip power, ROM, pain & activity of hand.	All but 2 cases had no limitation of daily activities, 4 cases reported mild pain. All ROM parameters in forearm and biplane wrist motion improved except for (1) case. Overall scores: 10 excellent & 4 good.	Malunion was reoperated successfully (1), refracture after fixator removal managed conservatively (1).
Masada:1989 [3]	Osteochondroma excision (12 ulnas & 2 radiuses), gradual ulnar lengthening with external fixator (3) & acute with bone graft (10), radial osteotomy (10), distal radius hemiepiphysiodesis (2), open reduction of dislocated radial head (2).	Radial articular angle, carpal slip, ulnar shortening. Relative radial shortening was measured for type III	Ulnar length was restored in all but one forearm. Radial articular angle & carpal slip improved in all cases.	ROM	Forearm rotation improved dramatically in all cases.	Recurrence of ulnar shortening (2), proximal radio-ulnar synostosis, transient radial nerve palsy (1 each).
Beutel:2014 [20]	Gradual ulnar lengthening with multiplanar external fixator (1).	Ulnar shortening, ulnar bowing, posterolateral radial head near dislocation were seen. MRI revealed entrapment of the annular ligament within the radiocapitellar joint, osteochondral impaction injuries of the anterior radial head, capitellum & injuries of the anterior trochlea and coronoid process.	Ulnar length & bow were restored & radial head relocated. MRI was not conducted postoperatively.	ROM & pain	Complete restoration of elbow ROM & resolution of pain. Forearm was unaffected preoperatively.	None
Hill:2011 [22] #	Osteochondroma excision, gradual ulnar lengthening (proximal diaphyseal), uniplanar, multiplanar Ilizarov or spatial/Ilizarov hybrid external fixator (5), & open reduction of radial head with neck osteotomy (1).	Radial articular angle, ulnar shortening, radial head dislocation	2 dislocated radial heads remained so, 1 dislocated & 1 subluxed after initially being located and 1 remained located before & after surgery.	Degree of deformity was recorded pre & postoperatively but No values or final outcomes were provided	NR	Poor callus regenerate successfully treated.#
Litzelmann:2012 [7]	In mild deformity: isolated osteochondroma excision (3 radiuses). Isolated radial osteotomies (2). In moderate deformity typically >11 y old: corrective distal 1/3 radial osteotomy with acute ulnar lengthening + bone grafting (3). In severe deformity: gradual ulnar lengthening over Intramedullary pin at diaphyso-metaphyseal junction, uniplanar external fixator (7) (4 with radial osteotomy & 3 with osteochondroma excision).	Radial articular angle, carpal slip, radial epiphyseal angle, ulnar variance, radial bowing & radial head dislocation assessed by Storen line.	*Non*-significant improvement of all radiologic parameter was noted. Two of the 5 dislocated radial heads preoperatively remained so postoperatively.	ROM, pain, Patient-reported functional assessment: QuickDASH.	ROM did not show statistically significant improvement. One out of the 3 patients with radial head instability & pain preoperatively remained so postoperatively and required a successful radial head resection at age 17 years. QuickDASH. Showed a significant improvement.	Revision surgeries at age 17y for deformity recurrence (2) (1 radial head resection & 1 radial osteotomy), fracture callus at 2 years postoperative (1).
Jiya:1997 [11]	Isolated osteochondroma excision (4), acute ulnar lengthening with screw fixation, osteochondroma excision (8), +/− radial osteotomy.	Linear axis, radial articular angle, carpal slip, ulnar shortening	Radial articular angle, carpal slip improved in most forearms or remained unchanged. Ulnar shortening was frequent.	NR (only chief complaints were recorded)	NR	Recurrence of ulnar shortening (5), reoperation for recurrent exostosis (1), reoperation for fracture/ non-union of callus to solid union (2).
Shin:2006 [10]	Isolated osteochondroma excision (11) (6 ulnas & 5 radiuses). Osteochondroma excision & ulnar lengthening (4) (2 gradual with uniplanar fixator & 2 acute), osteochondroma excision with Sauvé-Kapandji procedure (7).	Linear axis, radial articular angle, carpal slip, ulnar shortening	Isolated osteochondroma excision & ulnar lengthening: statistically *non*-significant improvement. Sauvé-Kapandji: Statistically significant improvement	ROM in forearm & elbow	Osteochondroma excision & ulnar lengthening: statistically *non*-significant improvement. Sauvé-Kapandji: Statistically significant improvement	36.4 % recurrence after simple osteochondroma excision (all required reoperations). Open reduction for persistently symptomatic radial head dislocation after ulnar lengthening (2). Reoperations for recurrent osteochondroma after Sauvé-Kapandji (2).
Rasool:2008 [31]	One-bone forearm (radioulnar fusion) (2).	Radial articular angle, carpal slip & ulnar shortening	Residual ulnar shortening in one forearm	ROM, grip strength	Both patients significantly improved in elbow, forearm rotation & grip strength	None
Pritchett:1986 [26]	Gradual mid-diaphyseal ulnar lengthening, uniplanar external fixator (6). Acute ulnar lengthening (4) including (2 with iliac crest graft and plate fixation & 2 over Rush rods). +/− radial osteotomy (5).	Radial articular angle, carpal slip, relative ulnar shortening,	Of the 6 subluxed/dislocated radial heads preoperative, 5 became stable postoperative.	ROM	ROM improved in most forearms	Recurrence of ulnar shortening (6), especially children & young adolescents, asymptomatic ulnar non-union & deep infection (1 each).
Massobrio:2015 [27]	Simultaneous gradual proximal ulna and distal radius lengthening with uniplanar external fixator (2).	Radial articular angle, carpal slip, ulnar shortening, relative ulnar shortening & radial length	Significant improvement in all measurements	Function, ROM & cosmetic	Significant improvement in ROM & function	None
Kelly:2016 [28]	Distal radius hemiepiphysiodesis (15).	Radial articular angle, carpal slip, ulnar tilt, lunate subsidence, metaphyseal epiphyseal angle	Statistically significant improvement in all 5 measurements except lunate subsidence	NR	NR	None
Tonogai:2015 [18]	Osteochondroma excision & interosseous membrane dissection, gradual ulna (2) or radial (1) lengthening, multiplanar Ilizarov or uniplanar fixator.	Radial articular angle, ulnar shortening	Improved	ROM	Improved	None
Refsland:2016 [14]	Gradual mid-diaphyseal Ulnar lengthening with uniplanar external fixator (17), +/− osteochondroma excision (14), +/− radial osteotomy (5).	Radial articular angle, carpal slip, radius of curvature, ulnar variance, angle of the radial and ulnar physes, elbow carrying angle, amount of radial head coverage	Statistically significant improvement in radius of curvature, ulnar variance, carrying angle, radial head coverage	ROM & pain	Statistically *non*-significant improvement in ROM & 5 patients who had pain preoperative had no pain postoperative.	Osteotomy for deformity recurrence (1), external fixator failure requiring exchange (2), premature consolidation (1).
Bauer:2017 [17]	Gradual ulna lengthening (1). Combined ulna & radius osteotomies (2)	Angular & rotational deformity of radius & ulna	Statistically significant improvement	ROM in rotation	Statistically significant improvement	Extensor pollicis Longus weakness (1).
Yang:2013 [34]	Reconstruction of the distal ulnar epiphysis by vascularized proximal fibula with epiphysis (2).	Radial articular angle, carpal slip & relative ulnar shortening	Significant improvement (persistent but asymptomatic radial head dislocation)	Function, pain ROM & cosmetic	Significant improvement	None
Bilen:2009 [23]	Osteochondroma excision, gradual ulnar lengthening multiplanar (2) or uniplanar (6) external fixator +/− radial osteotomy. Lengthening was Rush rods guided in (2) cases.	Radial articular angle, carpal slip & ulnar shortening	Significant improvement & all radial heads that were dislocated preoperative were reduced.	NR	Significant improvement but no goniometric measurements conducted	None
Mader:2003 [40]	Osteochondroma excision, gradual ulnar lengthening, uniplanar fixator (4).	Radial articular angle, carpal slip & ulnar shortening	Significant improvement	ROM, function.	Significant improvement	None
Peterson:2008 [32]	One-bone forearm (radioulnar fusion) (1).	Previous resection of distal ulna & radial head dislocation	Restoration of forearm length discrepancy.	ROM & stability in elbow	Significant improvement	None
Eralp:2016 [16]	Osteochondroma excision, gradual mid-diaphyseal ulnar lengthening, uniplanar external fixator (4) (over Steinman in 2 cases) and combined radius osteotomy.	Ulnar shortening, radial bow	Restoration of ulnar shortening & radial bow.	Daily life activities	Significant improvement	Recurrent radial deformity (1).
Waters:1997 [13]	Acute ulnar lengthening with plate fixation (17), osteochondroma excision (12) & radial osteotomy (11).	ulnar shortening, ulnar variance, radial inclination, radial articular angle, carpal slip (AP and lateral), forearm-third metacarpal angle, status of ossification of radial & ulnar physes, radial-head subluxation & congruence of the distal radioulnar joint.	Significant improvement	ROM	Most patients improved	Reoperations with bone graft for non/delayed union & broken plate (3), progressive radial head subluxation (1), annular ligament reconstruction (2), chronic elbow pain (2), creation of one-bone forearm (1), repeat lengthening (2).
Ip:2003 [30]	Osteochondroma excision, gradual ulnar lengthening, multiplanar or uniplanar external fixator or acutely with plating (6), & radial osteotomy (5) based on definite radiographic criteria.	Radial articular angle, carpal slip & ulnar shortening, relative ulna shortening	Significant improvement	ROM, subjective simple questionnaire to assess satisfaction	Significant improvement in ROM & all parents were satisfied with function & cosmetic.	Radial head dislocation during lengthening (1) was successfully reduced by pin stabilization.
Song:2013 [6]	Gradual ulnar lengthening with multiplanar external fixator & monofocal ulnar osteotomy (13), +/− radial osteotomy (5), +/− osteochondroma excision (4).	Radial articular angle, carpal slip & ulnar shortening, relative ulna shortening	Statistically significant improvements & the only dislocated radial head preoperative was reduced postoperative.	Subjective quality of life questionnaire, functional limitation of motion & pain	Most patients were satisfied with forearm appearance, had no pain on strenuous activities & could manage activities daily life activities easily.	Recurrence of osteochondroma & deformity (2), delayed union/nonunion (2).
Akita:2007 [8]	Isolated osteochondroma excision (13). Osteochondroma excision & ulnar lengthening (18) (8 gradual with uniplanar external fixator & 10 acute with bone graft), +/− gradual radial lengthening (4), +/− ulna osteotomy (2), +/− radial osteotomy (14), +/− open reduction radial head (2).	Radial articular angle, carpal slip & ulna variance	Statistically *non*-significant improvement of all measurements	Subjective quality of life questionnaire, pain, ROM & grip strength	Statistically *non*-significant improvement in ROM & grip strength. Most patients were pain free & had no restrictions of daily activities. Unsatisfactory cosmetic appearance (8).	Nonunion successfully treated with bone graft and internal fixation (3), fracture callus (2), temporary radial nerve paresis (1) & symptomatic radiocapitellar joint (2).
Matsubara:2006 [24]	Osteochondroma excision, gradual ulnar lengthening, uniplanar or multiplanar external fixator (7), +/− radial osteotomy (5), +/− gradual radius lengthening (2).	Radial articular angle, carpal slip, ulna variance & radial bow.	Recurrence of ulnar shortening in (5) cases. The other measurements improved moderately.	ROM & pain	Significant improvement in pain & forearm rotation except one case.	Recurrence of ulnar shortening (5) one of which was relengthened. Radial head subluxation (1).
Ishikawa:2007 [9]	Isolated osteochondroma excision (14) (6 from distal ulna & 8 from distal ulna + radius.	Radial articular angle, carpal slip, ulnar shortening, radial length, radial bowing.	Excision from distal ulna: statistically significant improvement in ulnar shortening, radial bow only. Excision from distal ulna + radius: *non*-significant improvement and/or deterioration in all measurements.	NR	NR	Osteochondroma recurrence of various degrees (7) (2 from ulna & 5 from ulna + radius).
Cheng:1991 [25]	Gradual ulnar lengthening with uniplanar external fixator without bone graft (4).	Ulnar length only	Satisfactory corrected	ROM, cosmetic	*Non*-significant improvement in ROM, but improved cosmetic & daily life activities.	None
Rodgers:1993 [33]	One-bone forearm (radioulnar fusion) with a pin or plate (2), +/− gradual lengthening & radial osteotomy (1).	Radial articular angle, carpal slip & ulnar shortening	Significant improvement except for residual ulnar shortening in (1) case.	ROM, activities of daily life	Satisfactory in elbow & wrist, returned to full-time manual occupation/competitive sports.	None
Irani:1993 [43]	Isolated osteochondroma excision from ulna & radius (8), gradual ulnar lengthening with uniplanar fixator (2) & bone graft with plating + radial osteotomy (1).	Ulnar shortening, & radial head subluxation/dislocation (3)	Relocated & asymptomatic	ROM	No improvement in forearm rotation	None
Fogel:1984 [12]	Isolated excision of the osteochondromas (ulna or radius) (12), acute ulnar lengthening & excision osteochondroma (2), acute ulnar lengthening, excision osteochondroma, & distal radius hemiepiphysiodesis (7). Fixations were with plate/rush rod, +/− bone graft.	Radial articular angle, carpal slip & ulnar shortening	Isolated excision: no improvement in radial articular angle & carpal slip. Ulnar lengthening & excision: no improvement. Ulna lengthening, excision & distal radius hemiepiphysiodesis: significant improvement.	ROM, pain & cosmetic.	Isolated excision: no improvement in neither rotation nor ulnar shortening but significant improvement in pain, no osteochondroma recurrence. Ulnar lengthening & excision: no improvement. Ulna lengthening, excision, & distal radius hemiepiphysiodesis: significant improvement.	None
Arms:1997 [29] ∞	Osteochondroma excisions (36), radial-head excisions (6), distal radius hemiepiphysiodesis (5), distal radial osteotomies (2), and ulnar lengthenings with external fixators (4). Combined procedures performed on a single patient in (11) occasions.	Radial articular angle, carpal slip, relative ulnar shortening, and forearm-third metacarpal angle.	Majority of patients demonstrated radiographic abnormalities	Telephone patient-reported questionnaire of quality of life	Majority of patients were in full-time jobs with minimal impact on activities of daily life.	NR

*N* number, *NR* not reported, *ROM* range of motion in forearm & elbow, +/− wrist, *VAS* visual analog scale, *SF-12* a quality of life scale that measures physical and mental components, *DASH* disabilities of the arm, shoulder and hand score.

μ clinicoradiologic results at final follow-up;

* only significant complications were mentioned;

† carpal slip could not be measured in 5 cases because the lunate was poorly ossified;

# mean age and follow-up, methodology, outcome and complications (except radial head status) were provided for 10 patients with various diagnostic groups and the details of HMO patients were not provided separately;

∞ the study comprised a cohort of patients treated conservatively and surgically 37 of which were subjected to a telephone questionnaire but patient demographics, disease characteristics and interventions were not provided separately for operated group.

**Figure 2:**
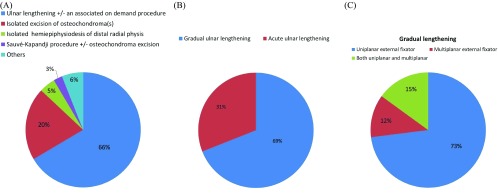
Comparative prevalence of the main interventions and techniques used in this review as percentages of forearms; (A) the main intervention groups used. Associated procedures include; osteochondroma excision, corrective radial osteotomy, distal radial hemiepiphysiodesis and open reduction of radial head. “Others” refers to isolated procedures as one-bone forearm, reconstruction of the distal ulnar epiphysis by vascularized proximal fibula epiphysis, radial osteotomy and lengthening; (B) gradual versus acute ulnar lengthening; (C) fixator choice of included studies.

Isolated excision of osteochondroma or at least without bone lengthening was conducted on 65 forearms (20.6%) of the summed number of forearms [7–12]. Temporary hemiepiphysiodesis of distal radial physis was performed on 29 forearms (9.2%) [3,12,28,29] one of which [28] 15 forearms (4.8%) was exclusively devoted to hemiepiphysiodesis. Sauvé-Kapandji procedure +/− osteochondroma excision was performed on seven forearms (2.2%) [10]. Creation one-bone forearm through radioulnar fusion was practiced on five forearms (1.6%) [31–33] and reconstruction of the distal ulnar epiphysis by vascularized proximal fibula including epiphysis was practiced on two forearms (0.5%) [34]. An open reduction of radial head +/− neck osteotomy was successfully performed on four forearms (1.3%) [3,10] and unsuccessfully on three forearms (1%) [8,22]. Resection of distal or proximal radioulnar synostosis was practiced on demand in two studies [15,21]. Radial head excision was not practiced in pediatric patients at least as a primary procedure. Proximal radio-ulnar fusion was practiced on one forearm to manage symptomatic radial head subluxation [12]. The comparative prevalence of the main interventions and techniques used in this review is demonstrated (Figures 2A, B, C). The overall complication rate was tolerable and showed no specific predilection for any of the main interventions employed in this review.

### Missing data

The included studies had missing data related to the following items: gender distribution (five studies) [10,11,16,28,29], number of forearms (one study) [29] and Masada subtype (seven studies) [12,17,22,25,26,28,29]. The overall skeletal burden of HMO was reported in only four studies [11,16,33,43] and detailed in one [27]. Hand dominance was reported in only four studies [19,31,33,39] and 11 studies used pain as an outcome measure. Previous surgeries were reported in four studies [13,16,27,32] and age of initial presentation in four [20,27,32,33]. Results of histopathologic examination were reported in six studies [16,20,24,27,34,41] and family history in six [3,8,20,30,33,34]. No study reported genetic testing and physiotherapy protocols were either poorly or not reported at all except for one study [22]. One study did not report the follow-up period as it was primarily designed to assess the utility of a computer simulation model [17]. One study reported patient ethnicity [28]. The study that practiced reconstruction of the distal ulnar epiphysis by vascularized proximal fibula did not report donor site morbidity [34]. One study reported conclusions that were discordant with the results [11]. The authors underscored the importance of ulnar lengthening in preventing progressive deformity and minimising functional disability despite reporting frequent deformity recurrence rates [11]. Grippingly, the authors employed neither objective nor subjective clinical outcome measures [11]. Generally speaking, we suggest that some of the missing data may have a potential impact on the validity of results and conclusions. For example failure to report the overall skeletal burden of HMO in terms of number and location and pain can influence the subjective patient-reported quality of life assessment [44–47]. Pain that was grossly underreported by the primary studies of this review has been found to be a major drive for surgery and negatively influenced by surgery [47]. The incidence of a positive family history in patients with HMO has been estimated to range from 62–96% [1]. Genotype-phenotype correlation studies demonstrated that certain types of gene mutations and the overall skeletal burden of HMO are associated with a worse clinical presentation particularly with respect to deformity and function [1,2,44,45,48–50]. This may actually have a predetermined impact on treatment outcomes.

## Discussion

### Summary of evidence

This systematic review included many chief surgical interventions each of which was multifaceted. For clarity and consensus, we will discuss each chief intervention separately. We believe that this practical approach will assist in answering our first research question. The most prevalent combination of surgical procedures encountered in this review was ulnar lengthening, +/− an associated procedure. Generally speaking, this surgical technique was adequately described and fairly constant across studies with a tolerable complication rate on the short-term. Nevertheless, there is poor evidence to demonstrate that the seemingly satisfactory results of many short-term studies are maintained on the intermediate-term [10] and more precisely on the long-term [7,8]. These long-term relatively sizable well-designed studies have questioned the value of surgical intervention even in association with deformity and radiographic abnormalities [7,8]. These studies correlated the clinicoradiologic outcomes with patients' self- reported functional outcome measures in children operated for HMO of forearm and argued for the choice of conservative treatment [7,8]. They found insufficient functional gains to justify surgery. In other words, preoperatively patients reported minimal functional impairment despite major clinical and radiologic abnormalities [7,8]. The only exception was symptomatic radial head dislocation. This discrepancy between the functional capacity and amount of forearm deformity was corroborated by three relatively sizable studies [22,24,29]. These findings have been confirmed by a natural history in a large cohort of untreated adult subjects with HMO [35].

Generally speaking, most studies tended to focus on the radiographic outcome measures at the expense of objective clinical parameters. Likewise, the objective physician-reported clinical outcome measures were implemented at the expense of the subjective patient-reported measures which were mostly non-validated and lacked in depth. Notwithstanding, these studies reported recognized cosmetic satisfaction of patients. Interestingly, in some studies patients were selected for surgery exclusively based upon radiographic criteria while clinical (objective or subjective) outcome measures were neither reported before nor after surgery [9,11,22,28]. It is noteworthy that complications such as recurrence of osteochondromas, and/or forearm deformity need longer durations to resurface especially in the skeletally immature population [9,24]. This undoubtedly overemphasizes the significance of conducting long-term follow-up studies and greatly undermines the quality of evidence extracted from such short-term studies. Additionally, this critically calls attention to the value of the validated subjective patient-reported overall quality of life scales. Using these scales has shown that HMO patients had lower scores compared to the general population [46,48]. We understand that a comprehensive assessment of patients' outcomes entails both subjective patient-reported and objective physician-reported instruments. In the light of such observations, it is important to rethink the cost/benefit profile of surgical intervention in paediatric HMO. In that regard, retrospective natural history studies may refine the wide and crude indications of surgery currently used in children with HMO. Two natural history studies are praised for aiming to identify radiographic predictors of radial head dislocation [4,51]. Likewise, well-designed randomized control studies where one group receives a definite surgical treatment and the other receives no treatment should be rethought. To satisfy the ethical demands of such studies, strict inclusion/exclusion criteria will have to be implemented before enrolment and randomization. Besides, adequate patient orientation in regard to risks and benefits of each treatment group and nature of the study will have to precede enrolment and randomization. Such study designs can generally yield valid results and generalizable conclusions. Nevertheless, we acknowledge the logistic and practical difficulties associated with such study designs. We believe that insufficient consensus about the indications of surgery and outcome scores in paediatric HMO is a major limitation of this systematic review. These discordant indications of surgery have also been noticed within individual study participants, further complicating evidence extraction. Likewise, the heterogeneity of the surgical procedures used, diversity in patient characteristics and relatively small study populations complicates evidence extraction. These above-mentioned features are inherent to retrospective studies that constituted the majority of included studies. Nonetheless, certain studies were remarkable for their methodologic quality. They implemented study designs that allow for control of selection and performance bias [6,8,10]. They neatly and separately reported the results of HMO patients in accordance with each distinct surgical procedure implemented. Additionally, some used assessor blinding methods and standardised surgeon related factors [6,8,10]. Isolated excision of osteochondroma can relief pain, satisfy cosmetic concerns and occasionally improve range of motion. Nevertheless, there is insufficient evidence for its use to initiate spontaneous deformity correction or improve overall limb function. In this review the cases of radial hemiepiphysiodesis, one-bone forearm, vascularized fibular graft, isolated radial osteotomies or lengthenings practiced as “stand alone” procedures were insufficiently prevalent to allow for meaningful conclusions. These procedures may be better suited to specific patient profiles and based upon surgeon's preference.

### Limitations and strengths

We acknowledge limitations of this review. The majority of the included studies were of low methodologic quality. Studies with low methodologic quality may impact negatively on outcome validity and conclusions. Narrative/traditional reviews are usually biased and subjective in contrast to systematic reviews which are often unbiased and objective [52]. The reliability and accuracy of systematic review recommendations should not be determined by methodologic quality of the primary studies included, but rather by the degree of methodological integrity implemented by researchers [52]. In this systematic review we formulated focused research questions that require specific answers. Additionally, we implemented a comprehensive review methodology that allowed for a reasonable control of bias. Therefore, we estimate that our comprehensive systematic review strategy can counterbalance the shortcomings of including studies with low methodologic quality. Of the 18 studies excluded on basis of language 11 were provisionally eligible for inclusion in this review. These 11 studies comprised 54 forearms. It is noteworthy that in three of these 11 studies the size of study population could not be accounted for due to missing or deficient abstracts. In general, the excluded studies were comparable to the included studies in terms of methodology and individual sample size. Hence, these language exclusions seem inconsequential.

### Conclusions

Ulnar lengthening +/− associated procedures can restore radiologic anatomy, improve appearance and to a lesser extent objective clinical parameters on the short/intermediate term. There is Poor evidence to demonstrate that these gains are maintained on the long-term. The impact of surgery on quality of life and function has not been adequately investigated. Considerable evidence suggests that surgery minimally impacts preoperative function. Predictors of surgical success in regard to patient and disease characteristics remain elusive. The complex interplay between the arrays of confounding variables has undermined the capability of most studies to provide well-grounded evidence to support and generalize their conclusions.

### Recommendations

- Comprehensive reporting of all actually and potentially relevant patient and disease characteristics that provide scope for determination of predictors of surgical success is prompted.
- Multicenter studies that allow for greater patient populations are encouraged because of disease rarity.
- Well-designed ethically tolerable prospective randomized “control” trials in carefully selected patient population groups should be considered.
- Validated quality of life assessment scales should be incorporated into patients' outcome measures.

## Conflict of interest

The authors Tamer A. EL-Sobky, Shady Samir, Ahmed Naeem Atiyya, Shady Mahmoud, Ahmad S. Aly and Ramy Soliman declare that they have no conflict of interest in connection with this article.
